# Effect of the Phenological Stage on the Phenolic Composition, and Antioxidant and Antimicrobial Properties of *Cirsium vulgare* (Savi) Ten. Extracts

**DOI:** 10.3390/life14091191

**Published:** 2024-09-20

**Authors:** Urte Griskeviciene, Justina Dambrauskiene, Mindaugas Marksa, Zaneta Mazeliene, Rimanta Vainoriene, Liudas Ivanauskas

**Affiliations:** 1 Department of Analytical and Toxicological Chemistry, Lithuanian University of Health Sciences, LT-50162 Kaunas, Lithuania; mindaugas.marksa@lsmu.lt (M.M.); liudas.ivanauskas@lsmu.lt (L.I.); 2 Department of Drug Chemistry, Medical Academy, Lithuanian University of Health Sciences, LT-50162 Kaunas, Lithuania; justina.dambrauskiene@lsmu.lt; 3 Laboratory of Biopharmaceutical Research, Institute of Pharmaceutical Technologies, Lithuanian University of Health Sciences, LT-50162 Kaunas, Lithuania; 4 Institute of Microbiology and Virology, Faculty of Veterinary, Lithuanian University of Health Sciences, LT-50161 Kaunas, Lithuania; zaneta.mazeliene@lsmu.lt; 5 Vilnius University Siauliai Academy Botanical Garden, LT-77175 Siauliai, Lithuania; rimanta.vainoriene@gmail.com

**Keywords:** phenological, antioxidant activity, antimicrobial activity, thistle, antioxidants

## Abstract

*Cirsium vulgare* (Savi) Ten. is a plant from the *Asteraceae* family that is commonly used in traditional medicine. The purpose of this work was to investigate the antioxidant and antimicrobial characteristics of phenolic compounds found in ethanol and dry extracts of *C. vulgare* leaves, inflorescence, and roots during various phenological stages. Apigenin-7-*O*-glucoside and chlorogenic acid were identified in practically all *C. vulgare* extracts. Extracts from leaves collected at the end of the phenological dormancy period and in the first growing year had the highest antioxidant (cupric ion-reducing antioxidant capacity of 12,938 Trolox equivalents/g dry weight) and antimicrobial activity (against *Staphylococcus aureus*, *Staphylococcus epidermidis*, *Pseudomonas aeruginosa*, *Proteus vulgaris*, and *Candida albicans*) with MIC values of ethanol extract from 16.7 mg/mL to 8.35 mg/mL. These extracts included a high concentration of chlorogenic acid and apigenin-7-*O*-glucoside. Also, dry extracts from *C. vulgare* roots and inflorescences showed a higher antimicrobial effect compared to ethanolic extracts with MIC values from 5.57 mg/mL to 3 mg/mL. The study emphasizes the critical role of phenological stages and raw material composition in the accumulation of phenolic compounds and their biological activity in *C. vulgare*. The findings suggest that extracts from *C. vulgare* leaves, especially those collected at the end of the phonological dormancy period, are promising candidates for further research into bioactive compounds with potential medicinal applications. The strong antioxidant and antibacterial properties of these extracts highlight their potential for development into natural pharmaceutical products.

## 1. Introduction

*Cirsium* is characterized by around 450 species globally, with 200 found in diverse regions such as Asia, Central America, Europe, North Africa, and North America [1]. *C. vulgare*, also known as spear thistle, bull thistle, or common thistle, is a highly invasive plant that originated in Eurasia and has successfully spread to every continent except Antarctica [2]. *C. vulgare* (Savi) Ten. is a distinctive biennial plant in the *Asteraceae* family, growing as a branching, erect biennial reaching 2 to 6 feet in height. Rosettes form in the first growing year, followed by flowering stems in the second. The plant also has long, sharp spines on the leaves at the midrib and the tips of the lobes. Their leaves are deeply lobed and hairy—there are coarse hairs on leaf tops, making the leaf feel rough to the touch, and wooly hairs on the underside. Leaf bases extend down onto stems and form spiny wings along the stems. It is also easy to identify it from its pink-magenta flower head top because each stem and flower head are “gumdrop” shaped, and spines extend all around the base of the flower heads [3,4].

*C. vulgare*, which is considered one of the most prolific invasive species in the world [5] has not been researched much and has only been used in folk medicine until now. Traditionally used in folk medicine, this plant has diverse potential uses, including treating inflammations, liver and pancreas diseases [6], stomach pains, and other chronic conditions. Ongoing research aims to identify the active compounds in the plant and their medicinal properties for potential modern medicinal applications [7]. *C. vulgare* has a rich history of traditional uses, including being used as an anxiolytic in certain regions, particularly in Poland [8]. In Polish folk medicine, it is also employed as a diuretic, astringent, and anti-inflammatory agent [9]. In addition, C. *vulgare* is being cultivated in gardens to attract birds and butterflies [10]. However, data suggests that antioxidant and antimicrobial effects characterize this plant [11].

The major phytoconstituents present in various species of *Cirsium* are flavonoids, steroids, alkaloids, terpenes, triterpenes, sesquiterpenes, cyclic ether, fatty acids, sterols, glycerol, aldehydes, ketones, phenolic acids, and essential oil components [1]. The biological effects are likely due to the chlorogenic acid, apigenin-7-*O*-glucoside, rutin, hyperoside, isoquercitrin, and other phenolic compounds, especially chlorogenic acid and apigenin-7-*O*-glucoside, most of which are identified. There is data in the literature that chlorogenic acid possesses anti-inflammatory, antioxidant, cardioprotective, hepatoprotective, neuroprotective, anticancer, antiviral, and antimicrobial effects [12]. Meanwhile, apigenin-7-*O*-glucoside possesses antibacterial activity, especially against *S. aureus* and *E. faecalis* [13]. Also, apigenin-7-*O*-glucoside has anti-inflammatory, antioxidant, and anticancer properties [14].

The biosynthesis and accumulation of secondary metabolites in plants, namely phenolic compounds, which are characterized by biological effects, are influenced by several factors, including species specificity, duration of vegetation, and environmental conditions (such as climate, altitude, and soil characteristics) [15]. Although phenolic compounds are generated throughout the plant, their concentration in different organs changes during growth and development. This is primarily due to changes in gene expression that affect enzyme activity in phenolic compound production [16]. Since thus far there are no published reports concerning the effect of the phenological stage on the accumulation of biologically active compounds and the antioxidant and antimicrobial potentials of *C. vulgare* extracts, this study was undertaken. This study is aimed at determining the effect of different phenological stages, the change in the number of active compounds in different plant parts that have been accumulated by the plant according to the stages, and the antioxidant and antimicrobial effects of the selected extracts according to the active compounds. This is a large-scale study, as several sections of the plant (roots, leaves, and inflorescences) were collected throughout a two-year period.

## 2. Materials and Methods

### 2.1. Plant Material

*Cirsium vulgare* (Savi) Ten. samples were collected from the Siauliai Academy Botanical Garden for research purposes. The botanical garden’s geographical coordinates are 55°55′57″ N, 23°16′59″ E (WGS). The study focused on the leaves, inflorescences, roots, and seeds of the *C. vulgare* plants, collected over two years during phenological periods ranging from the end of the dormant period to seed maturity. Each sample was collected from three different plant parts; all experiments were repeated 3 times (*n* = 3). Extract number meanings are added to Table A1. Each part of the plant was dried separately at 40 °C in a drying chamber and then ground into a fine powder using an Ultra Centrifugal Mill ZM 200 (Retsch, Hann, Germany) with a 0.5 mm trapezoid hole sieve. The herb’s final moisture content was 7.37 ± 0.3%.

### 2.2. Chemicals

Ethanol 96% (*v*/*v*) (CAS 64-17-5) from Vilniaus degtinė in Vilnius, Lithuania, was used. The water for sample preparation was produced using a Super Purity Water System from Millipore in the USA. HPLC-grade and analytical-grade reagents were used in the study. These reagents included trifluoroacetic acid (CAS 76-05-1) and acetonitrile (CAS 75-05-8) from Sigma Aldrich in Steinheim, Germany. Standards of various compounds were also used, including chlorogenic acid (CAS 327-97-9), apigenin-7-*O*-glucoside (CAS 578-74-5), apigenin (CAS 520-36-5), luteolin (CAS 491-70-3), hyperoside (CAS 482-36-0), neochlorogen (CAS 906-33-2), isoquercitrin (CAS 482-35-9), p-coumaric acid (CAS 501-98-4), caffeic acid (CAS 331-39-5), trans-ferulic acid (CAS 99 537-98-4), and 4-*O*-caffeoylquinic acid (CAS 905-99-7). These standards were also obtained from Sigma Aldrich in Steinheim, Germany. Additionally, Cuprum (II) chloride dihydrate (CAS 10125-13-0) was purchased from Alfa Aesar GmbH & Co KG in Karlsruhe, Germany, while neocuproin (CAS 484-11-7) was obtained from Sigma-Aldrich Chemie in Steinheim, Germany. The buffer system used in the study was ammonium acetate (CAS 631-61-8) from Sigma-Aldrich in Overijse, Belgium. The referent standard Trolox (98%) (CAS 53188-07-1) was purchased from FlukaChemika in Buchs, Switzerland. Mueller–Hinton agar, used in the study, was obtained from BBL in Baltimore, MD, USA.

### 2.3. Production of Ethanolic and Dry Extracts from C. vulgare

To produce an ethanolic extract, the heat-reflux extraction method was used. Firstly, we took 0.1 ± 0.001 g of dried and milled leaves, inflorescence, and roots and mixed them with 50% ethanol in a 250 mL round bottom flask. Then, the flask was placed under reflux for 1.5 h at 90 °C. The resulting extract was filtered through a 0.22 μm PVDF syringe filter. The same steps were followed for producing dry extracts, but the end products were made using ethanolic extracts with a vacuum to evaluate the antimicrobial effect of dry extracts. The goal was to eliminate the ethanol. After removing the ethanol, the solutions were placed in a dryer (40 °C) to obtain a dry mass.

### 2.4. Qualitative and Quantitative Analysis of the HPLC Method

Qualitative and quantitative analysis was carried out on a Waters Alliance 2695 liquid chromatograph equipped with a Waters 996 photodiode array detector (PDA) and an ACE C18 (250 mm × 4.6 mm × 5 μm) column (Advanced Chromatography Technologies, Aberdeen, Scotland) [17]. The mobile phase consisted of solvents A (trifluoroacetic acid (0.1%)) and B (acetonitrile). The linear gradient elution profile was as follows: 95% A/5% B at 0 min, 85% A/15% B at 8 min, 80% A/20% B at 30 min, 60% A/40% B at 48 min, 50% A/50% B at 58–65 min, 5% A/95% B at 66–70 min, and 95% A/5% B at 71 min. The flow rate was 1 mL/min, and the injection volume was 10 μL. Absorption was measured in the range from 330 nm to 360 nm. The detection of phenolic compounds was performed using the following standards: chlorogenic acid, apigenin-7-*O*-glucoside, apigenin, luteolin, hyperoside, neochlorogenic acid, isoquercitrin, *p*-coumaric acid, caffeic acid, trans-ferulic acid, and 4-*O*-caffeoylquinic acid.

### 2.5. Measurement of Antioxidant Activity by CUPRAC Assay

An experiment on sample reduction was carried out using the spectrophotometric CUPRAC method [18]. This method uses cupric ion-reducing antioxidant capacity (CUPRAC) and electron transfer (ET) in vitro assays. CUPRAC reagent solution is mixed with copper chloride (CuCl_2_x2H_2_O), neocuproin (CH_3_)_2_C_12_H_6_N_2_), and ammonium acetate buffer (pH = 7) solution in 1:1:1 to obtain a total reaction mixture. This mixed solution stays in the dark, at room temperature, for one hour. There were used extracts for this measurement with 0.02 mg/mL concentration. After that, 10 μL of sample extract was mixed with 3 mL of distilled water, followed by the addition of copper chloride, neocuproin, and ammonium acetate buffer (pH = 7) solution. After 30 min of incubation at room temperature, absorbance was recorded at 450 nm. Results were expressed as μM Trolox calibration curve y = 0.00004x + 0.0215; R^2^ = 0.9991.

### 2.6. Determination of Antimicrobial Activity

The antimicrobial activity was assessed using the dilution method on solid agar nutrient media, specifically Mueller–Hinton agar (Mueller–Hinton II agar, BBL, Cockeysville, ML, USA) [19]. The following reference strains were utilized in the study: *Staphylococcus aureus* ATCC 2592, *Staphylococcus epidermidis* ATCC 12228, *Enterococcus faecalis* ATCC 29212, *Escherichia coli* ATCC 25922, *Klebsiella pneumoniae* ATCC 13883, *Pseudomonas aeruginosa* ATCC 27853, *Bacillus cereus* ATCC 11778, *Candida albicans* ATCC 10231, and *Proteus vulgaris* ATCC 8427.

The non-spore-forming bacteria (*Staphylococcus aureus*, *Staphylococcus epidermidis*, *Enterococcus faecalis*, *Escherichia coli*, *Klebsiella pneumoniae*, *Pseudomonas aeruginosa*, and *Proteus vulgaris*) were grown for 20–24 h at 35–37 °C on Mueller–Hinton agar. Bacterial suspensions were prepared from these cultures in sterile physiological sodium chloride (0.9%) solution, standardized using McFarland’s standard indicator to a turbidity equivalent to a 0.5 McFarland standard, indicating 1.5 × 10^8^ cells/mL. *Bacillus cereus*, a spore-forming bacterium, was cultured for seven days at 35–37 °C on Mueller–Hinton agar. Post-incubation, the spores were harvested, heated at 70 °C for 30 min, and diluted with saline to achieve a concentration of 10 × 10^6^ to 100 × 10^6^ spores/mL. The fungal reference strain *Candida albicans* was grown for 20–24 h at 30 °C on Sabouraud agar, and yeast suspensions were similarly standardized using McFarland’s standard indicator.

In this study, the antimicrobial effects of 14 ethanolic extracts of *Cirsium vulgare* (from different phenological stages and plant parts) were investigated, along with two dry extracts made from inflorescences and roots at various phenological stages. The concentrations of *C. vulgare* ethanol extracts used were as follows: U1–U10 at 0.1 mg/mL, U11–U13 at 0.05 mg/mL, and U13* at 0.02 mg/mL. All dry extract concentrations (UF, UR) were standardized to 0.033 mg/mL. Initially, 1 mL of the *C. vulgare* ethanol extract was added to sterile Petri dishes labeled U1–U13, while 0.5 mL of thistle extract was added to the U13* Petri dish. Dry extracts (UR, UF) were added in varying volumes (1 mL, 0.5 mL, 0.4 mL, 0.3 mL, 0.2 mL, and 0.1 mL) to sterile Petri dishes. Subsequently, 5 mL of liquid Mueller–Hinton agar, pre-cooled to 45 °C, was added to each Petri dish and mixed with the sample volumes. Once the agar solidified, each segment was inoculated with the prepared suspensions of reference microorganisms.

The inoculated Mueller–Hinton agar plates were incubated at 35 °C for 20–24 h in a thermostat, followed by an additional 24 h at room temperature. The antimicrobial activity of each tested sample was determined by comparing it to the growth of reference microorganisms. If the reference microorganism grew on the culture site, the sample did not exhibit inhibitory activity. Conversely, if no growth was observed, the sample demonstrated antimicrobial activity against the reference microorganism. The Minimum Inhibitory Concentration (MIC) of each sample was determined using the agar dilution method, defined as the lowest concentration of the antimicrobial agent that inhibits visible growth of the microorganism after overnight incubation. The antimicrobial activity of ethanol extracts and dry extracts from *C. vulgare*, along with antibiotics including penicillin, ciprofloxacin, ampicillin, amoxicillin, and daptomycin, was evaluated.

### 2.7. Data Analysis

The quantitative results are presented as mean ± standard deviation (SD) of 3 replicates. The data were processed using Microsoft Office Excel 2023 (Microsoft, Redmond, WA, USA) and SPSS 25 (IBM, Armonk, NY, USA) software. The significant differences between extracts of *C. vulgare* inflorescence, leaves, and roots were calculated by one-way ANOVA, followed by Tukey’s post hoc comparison test. Pearson’s correlation analysis was performed to elucidate the relationship between the content of phenolic compounds and the antioxidant capacity of inflorescence, leaves, and root extracts. Statistical data analysis for antibacterial activity testing was conducted using ANOVA and Tukey’s HSD post hoc test. The statistical data analysis was performed by applying the ANOVA with Tukey’s HSD post hoc test for antibacterial activity testing. In all cases, differences were considered statistically significant when *p* < 0.05.

## 3. Results

### 3.1. Phenological Analysis of Phenolic Compounds

**Figure 1:**
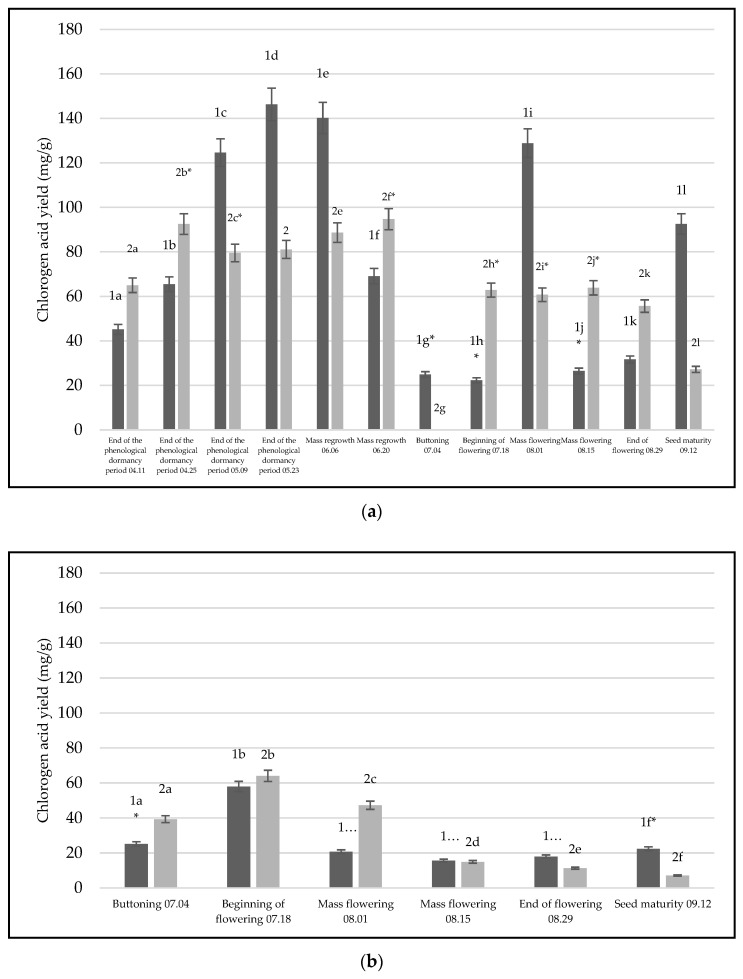

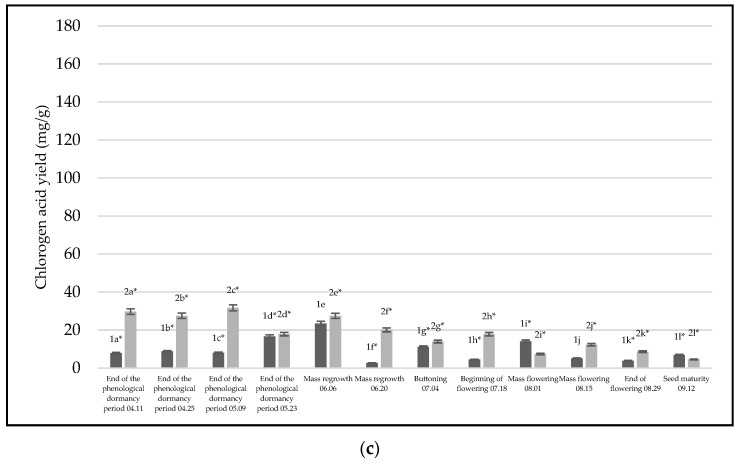
Yields of the phenolic compound—chlorogenic acid—detected in the raw extracts of thistle for two years, 2021–2022: (**a**) extracts made from leaves, (**b**) extracts made from inflorescence, (**c**) extracts made from plant roots. The black color illustrates 2021 materials, gray—2022 materials. Different letters indicate statistically significant differences between *C. vulgare* phenological stages samples (*p* < 0.05). The asterisks (*) indicate that the compared groups have one insignificant difference between them *p* > 0.05.

**Figure 2:**
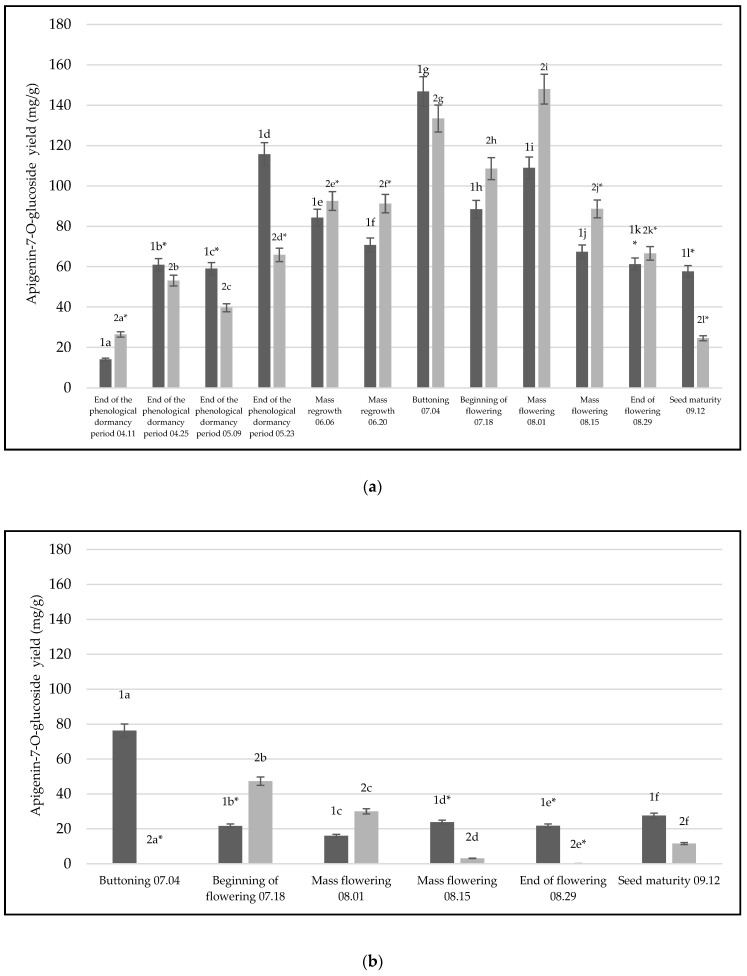

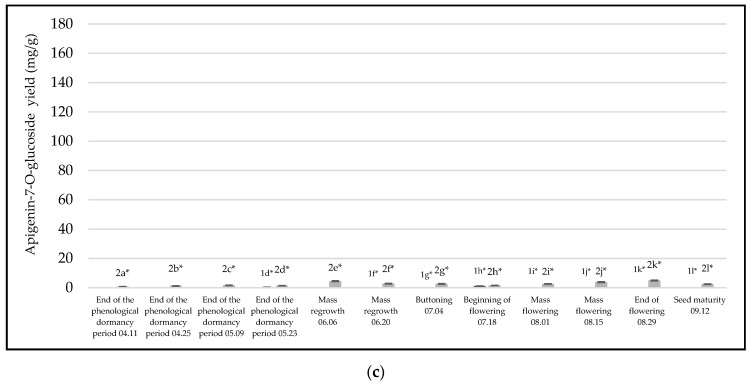
Yields of the phenolic compound—apigenin-7-*O*-glucoside—detected in the raw extracts of thistle for two years, 2021–2022: (**a**) extracts made from leaves, (**b**) extracts made from inflorescence, (**c**) extracts made from plant roots. The black color illustrates 2021 materials, gray—2022 materials. Different letters indicate statistically significant differences between *C. vulgare* phenological stages samples (*p* < 0.05). The asterisks (*) indicate that the compared groups have one insignificant difference between them *p* > 0.05.

The HPLC-PDA method was validated and used to identify eleven main phenolic compounds in extracts. Table A2 provides validation data. A comparison of yields for active compounds in bull thistle’s raw materials for two years (2021–2022) of phenological growth is presented in Figure 1 and Figure 2 for the compounds with the highest yields. There, the chlorogenic acid yields in two-year raw material extracts were presented (Figure 1 and Figure A3). After comparing the extracts made from different raw materials over two years, it was discovered that the extracts made from *C. vulgare* leaves yielded significantly higher results in comparison to the inflorescence (*p* = 0.0017, *p* < 0.05) and root (*p* = 0.0001, *p* < 0.05) extracts of the first year of growth (Figure 1a). Additionally, extracts made from first-growing-year inflorescence (Figure 1b) and roots (Figure 1c) have not shown significant differences (*p* = 0.258, *p* > 0.05). Second-growing-year extracts made from different raw materials showed the same tendency; comparing second-growing-year leaves with inflorescence and root extracts, a significant difference between these materials was found additionally with inflorescence (*p* = 0.00011, *p* < 0.05) and roots (*p =* 0.00014, *p* < 0.05) (Figure 1a). Compared to the first growing year, the second-growing-year inflorescence (Figure 1b) compared to roots (Figure 1c) did not show significant differences (*p* = 0.359, *p* > 0.05). Furthermore, comparing extracts made from *C. vulgare* leaves, this research showed that the highest yields of chlorogenic acid were found in extracts of first-year materials at the end of the phenological dormancy period (yield 146.28 mg/g) and mass regrowth (yield 140.15 mg/g). Furthermore, these phenological stages have shown significant differences between groups of other first-year phenological stages (*p* = 0.0001, *p* < 0.05) (Figure 1a). The highest concentration of chlorogenic acid in inflorescence extracts was found during the second growing year at the beginning of the flowering stage (yield—64.083 mg/g). Significant differences were observed between this phenological stage and other stages in the groups (*p* = 0.0001, *p* < 0.05). The highest yields of chlorogenic acid in root extracts were detected in the second growing year of roots at the end of the phenological dormancy period stage (yield—31.71 mg/g) (Figure 1c). Moreover, this phenological stage compared with others has shown significant differences between the groups of other stages (*p* = 0.0001, *p* < 0.05), except for one stage compared to the end of the dormancy period 11 April (*p* = 0.428, *p* > 0.05). Significant differences were also found between the two years of material (Figure 1c). First-growing-year extracts yielded significantly more chlorogenic acid than second-growing-year materials. (*p* = 0.039, *p* < 0.05).

One of the highest yields in the raw materials of both comparative years was apigenin-7-*O*-glucoside, which was also isolated in the extracts (Figure 2). Comparing two years of extracts made from different raw materials, it was found that extracts made from *C. vulgare* leaves (Figure 2a) had significantly higher yields compared to inflorescence (*p* = 0.0004, *p* < 0.05) (Figure 2b) and root (*p* = 0.0172, *p* < 0.05) (Figure 2c) extracts of the first growing year. Additionally, extracts made from the first-growing-year inflorescences and roots also showed significant differences (*p* = 0.0003, *p* < 0.05) (Figure 2b). Second-growing-year extracts made from different materials showed the same tendency; comparing second-growing-year leaves with inflorescences and root extracts, it was found that there is a significant difference between these materials, also with inflorescences (*p* = 0.00013, *p* < 0.05) (Figure 2b) and roots (*p =* 0.00017, *p* < 0.05) (Figure 2c). Statistical analysis did not reveal significant differences between second-growing-year inflorescences (Figure 2b) and roots (Figure 2c) (*p* = 0.138, *p* > 0.05). Furthermore, comparing two growing years of materials, only extracts made from *C. vulgare* roots showed significant differences within two years of material yield (*p* = 0.00017, *p* < 0.05) (Figure 2c). Also, this research has shown that the highest yields of apigenin-7-*O*-glucoside were found in leaf extracts of the second growing year at the mass flowering phenological stage (yield 147.96 mg/g) (Figure 2a). The highest yields of apigenin-7-*O*-glucoside in inflorescence extracts were detected in the first growing year of inflorescences in the buttoning stage (yield—76.30 mg/g). There were significant differences observed between this particularly phenological stage and other groups of stages (*p* = 0.0001, *p* < 0.05) (Figure 2b). The highest yields of apigenin-7-*O*-glucoside were detected in the second growing year of roots during the mass regrow stage (yield—4.484 mg/g). Moreover, this phenological stage has shown significant differences between groups of other stages (*p* = 0.0001, *p* < 0.05), except for one stage compared to the end of flowering 08.29 (*p* = 0.982, *p* > 0.05) (Figure 2c). Significant differences were found between the second-growing-year phenological stages and the mass flowering phenological stage (*p* = 0.0001, *p* < 0.05).

After analyzing the raw materials from the two years (2021–2022), extracts with the highest yields of phenolic compounds were selected for further research. The selected extracts for further investigations made from roots collected at the end of the phenological dormancy period (11 April), roots collected at the end of the phenological dormancy period (25 April), roots collected at the end of the phenological dormancy period (9 May), leaves collected at the end of the phenological dormancy period (25 April), leaves collected during the mass regrowth period (6 June), inflorescences collected during the mass flowering period (30 July), leaves collected during the seed maturity period (12 September), inflorescences collected during the seed maturity period (12 September), leaves collected at the end of the phenological dormancy period (25 April), leaves collected at the end of the phenological dormancy period (23 May), leaves collected during the mass regrowth period (6 June).

### 3.2. Antioxidant Activity of C. vulgare Extracts

**Figure 3:**
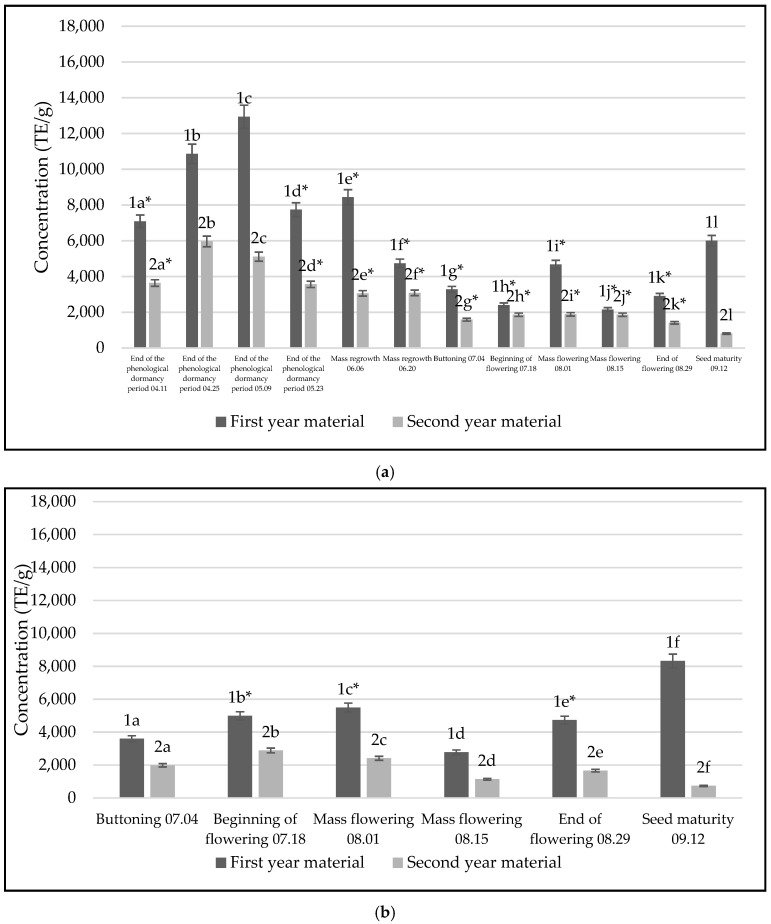

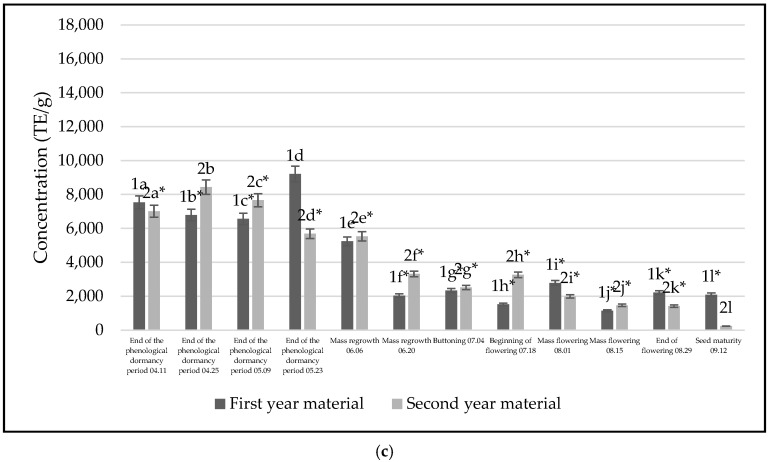
Antioxidant activity of *C. vulgare* extracts made from the raw materials for two years, 2021–2022, their comparison: (**a**) extracts made from leaves, (**b**) extracts made from inflorescence, (**c**) extracts made from plant roots. The black color illustrates 2021 materials, gray—2022 materials. Different letters indicate statistically significant differences between *C. vulgare* phenological stages samples (*p* < 0.05). The asterisks (*) indicate that the compared groups have one insignificant difference between them *p* > 0.05.

We studied the antioxidant effect of two-year-old raw materials, as the plant is biennial. We needed to determine which phenological stage of the plant and which extracts of the raw materials had the highest antioxidant effect, just as we did with the data on the accumulation of phenolic compounds. As can be seen in Figure 3a, extracts made from plant leaves had the highest antioxidant activity compared to extracts made from inflorescences and roots. The highest antioxidant activity (12,938 TE/g DW) was determined in the extracts from *C. vulgare* leaves, collected at the end of the phenological dormancy period and at the first growing year. Additionally, the smallest antioxidant activity was determined in the extracts from *C. vulgare* leaves, collected at seed maturity (813 TE/g DW) in the second growing year (Figure 3a). Significant differences were additionally found in first-growing-year materials with inflorescences (*p* = 0.024, *p* < 0.05) and roots (*p* = 0.0046, *p* < 0.05). Also, second-growing-year leaves had the highest antioxidant activity compared to inflorescence (*p* = 0.0126, *p* < 0.05) and roots (*p* = 0.0047, *p* < 0.05).

Comparing the raw materials from two years, the first-growing-year inflorescences had significantly higher concentrations than the second-growing-year inflorescences (*p* = 0.002, *p* < 0.05). The highest antioxidant activity (8331 TE/g DW) was determined in the extracts from *C. vulgare* inflorescences, collected at the seed maturity phenological stage and the first growing year. Additionally, the smallest antioxidant activity was determined in the extracts from *C. vulgare* inflorescences, collected at seed maturity (738 TE/g DW) in the second growing year (Figure 3b).

When comparing the antioxidant activity of the root extracts between the two-year raw materials, it was found that only the extracts produced with the second-growing-year raw materials showed more potent antioxidant activity. Still, the results had no significant differences (*p* = 0.431, *p* > 0.05). The highest antioxidant activity (9213 TE/g DW) was determined in the extracts from *C. vulgare* roots, collected at the end of the phenological dormancy period and at the first growing year. Additionally, the smallest antioxidant activity was determined in the *C. vulgare* root extracts, collected at seeds maturity (238 TE/g DW) in the second growing year (Figure 3c).

After a two-year (2021–2022) analysis of the raw materials’ antioxidant activity, the extracts selected for further research were chosen based on the obtained antioxidant concentrations. Selected extracts for further investigations were made from roots collected at the end of the phenological dormancy period (11 April), roots collected at the end of the phenological dormancy period (25 April), roots collected at the end of the phenological dormancy period (9 May), leaves collected at the end of the phenological dormancy period (25 April), leaves collected during the mass regrowth period (6 June), inflorescences collected during the mass flowering period (30 July), leaves collected during the seed maturity period (12 September), inflorescences collected during the seed maturity period (12 September), leaves collected at the end of the phenological dormancy period (25 April), leaves collected at the end of the phenological dormancy period (23 May), leaves collected at mass regrowth period (6 June).

### 3.3. Antimicrobial Activity of C. vulgare Extracts

**Figure A1:**
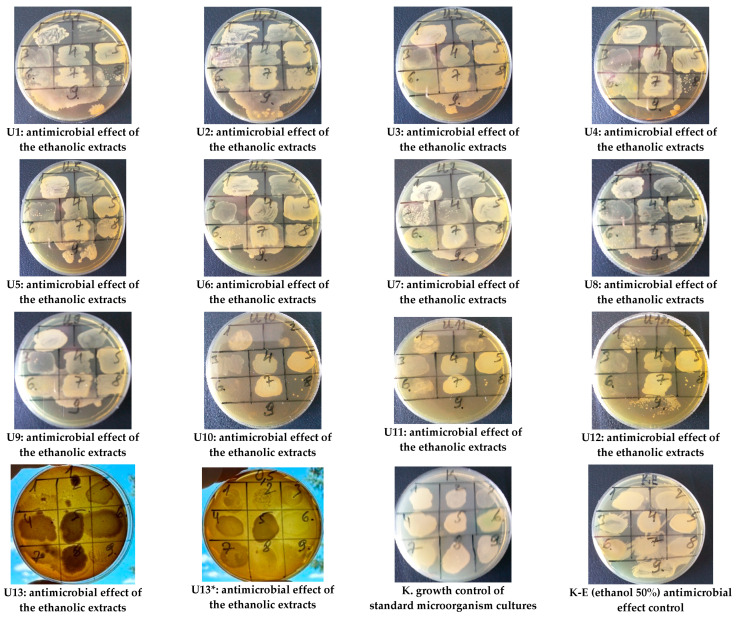
Antimicrobial activity using dilution method on solid nutrient media—Mueller–Hinton agar. C. vulgare ethanolic extracts visualization of the antimicrobial effect on the growth of reference microorganism cultures. Extracts number meaning: U1: roots from the end of the phenological dormancy period (11 April 2022), U2: roots from the end of the phenological dormancy period (25 April 2022), U3: roots from the end of the phenological dormancy period (9 May 2022), U4: leaves from the end of the phenological dormancy period (25 April 2022), U5: leaves from the end of the phenological dormancy period (23 May 2022), U6: leaves from mass regrowth period (6 June 2022), U7: inflorescence from mass flowering period (30 July 2021), U8: leaves from seeds maturity period (12 September 2021), U9: inflorescence from seeds maturity period (12 September 2021), U10 (0.3 g raw materials + 30 Ml ethanol); U10: leaves from the end of the phenological dormancy period (25 April 2022), U11: leaves from the end of the phenological dormancy period (23 May 2022), U12: leaves from mass regrowth period (6 June 2022) (1 g raw materials + 20 Ml ethanol); U13: leaves from the end of the phenological dormancy period (23 May 2022) (2 g raw materials + 10 Ml ethanol); U13*: leaves from the end of the phenological dormancy period (23 May 2022) (2 g raw materials + 10 Ml ethanol). Segment 1—Staphylococcus aureus ATCC 25923; Segment 2—Staphylococcus epidermidis ATCC 12228; Segment 3—Enterococcus faecalis ATCC 29212; Segment 4—Escherichia coli ATCC 25922; Segment 5—Klebsiella pneumoniae ATCC 13883; Segment 6—Pseudomonas aeruginosa ATCC 27853; Segment 7—Bacillus cereus ATCC 11778; Segment 8—Candida albicans ATCC 10231; Segment 9—Proteus vulgaris ATCC 8427.

As can be seen in the table below, the ethanolic extracts added in the amount of 1 mL to 5 mL of agar medium with inoculated cultures were the most active against the fungus *Candida albicans* (extracts U11, U12, which MIC 8.35 mg/mL), Gram-negative *Proteus vulgaris* (extracts U11, U12, MIC 8.35 mg/mL), and Gram-positive *Staphylococcus epidermidis* (extracts U12, U13, MIC 8.35 mg/mL) cell cultures. Ethanolic extracts had very little activity against Gram-positive *Staphylococcus aureus* (extract U13, MIC 8.35 mg/mL) and Gram-negative *Pseudomonas aeruginosa* (extract U10, MIC 16.7 mg/mL). Thistle ethanolic extracts had no antimicrobial activity against Gram-positive *Enterococcus faecalis*, Gram-negative *Escherichia coli*, Gram-negative *Klebsiella pneumoniae,* and Gram-positive *Bacillus cereus*. Overall, at lower concentrations, these ethanolic extracts did not affect any of the microorganism lines at all (Table 1). The visualization of conducted research is presented in Appendix A, Figure A1.

**Figure A2:**
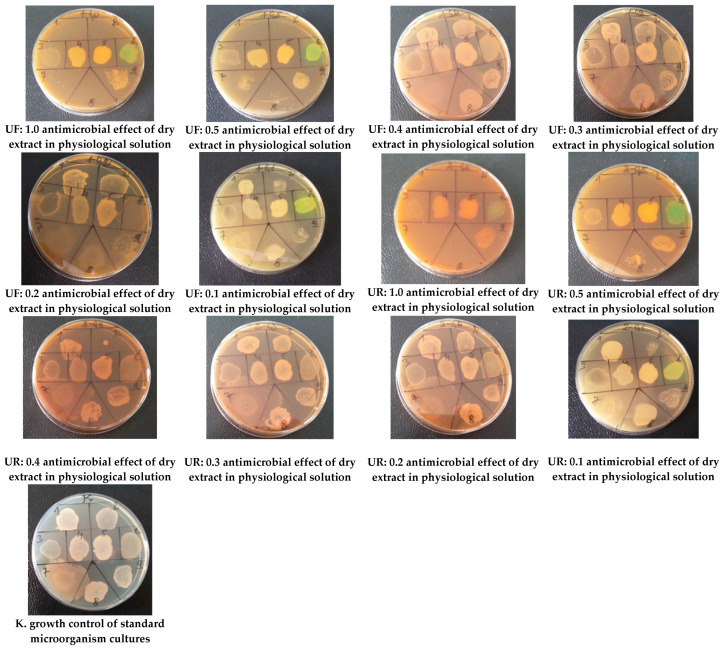
Antimicrobial activity using the dilution method on solid nutrient media—Mueller–Hinton agar. C. vulgare dry extract visualization of the antimicrobial effect on the growth of reference microorganism cultures. Extracts letters meanings: UF1-C. vulgare inflorescence from the seed’s maturity period, UR-C. vulgare roots from the end of the phenological dormancy period. Segment 1—Staphylococcus aureus ATCC 25923; Segment 2—Staphylococcus epidermidis ATCC 12228; Segment 3—Enterococcus faecalis ATCC 29212; Segment 4—Escherichia coli ATCC 25922; Segment 5—Klebsiella pneumoniae ATCC 13883; Segment 6—Pseudomonas aeruginosa ATCC 27853; Segment 7—Bacillus cereus ATCC 11778; Segment 8—Candida albicans ATCC 10231; Segment 9—Proteus vulgaris ATCC 8427.

**Figure A3:**
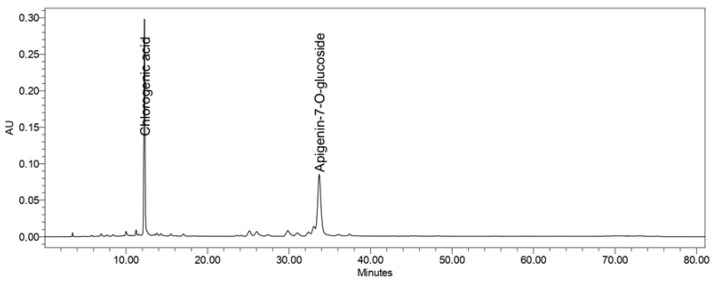
HPLC-PDA profiles of chlorogenic acid and apigenin-7-O-glucoside at 320 nm.

In contrast to the ethanolic extracts of *C. vulgare* (Savi) Ten., dry extracts from the same source exhibited varying effects on the tested cell lines. Dry extract UF demonstrated efficacy against Gram-positive cell lines, including *Staphylococcus aureus* (MIC 3 mg/mL), *Staphylococcus epidermidis* (MIC 3 mg/mL), *Bacillus cereus* (MIC 3 mg/mL), and *Proteus vulgaris* (MIC 5.567 mg/mL) at concentrations ranging from 1 mL/5 mL agar to 0.5 mL/5 mL agar. However, it showed no activity against *Proteus vulgaris* at concentrations of 0.5 mL/5 mL agar or below and against the other mentioned cultures at concentrations of 0.4 mL/5 mL or below agar. On the other hand, another extract, UR, displayed activity against the same bacterial cultures and exhibited higher efficacy against Gram-positive *Staphylococcus epidermidis* (MIC 2.47 mg/mL) at a concentration of 0.4 mL/5 mL agar, as outlined in Table 2. The visualization of the conducted research is presented in Appendix A, Figure A2.

Also, a comparative study with antibiotics was performed for three reference cell lines—*Staphylococcus aureus* ATCC 25923, *Staphylococcus epidermidis* ATCC 12228, and *Pseudomonas aeruginosa* ATCC 27853 (Table A3). The antimicrobial activity was evaluated against key antibiotics, including Ampicillin, Gentamycin, Tetracycline, and Ciprofloxacin, targeting both Gram-positive and Gram-negative bacteria. Against Gram-positive bacteria *Staphylococcus aureus* and *Staphylococcus epidermis,* all tested antibiotics had strong activity: Ampicillin (MIC ≥ 0.5 μg/mL), Gentamycin (MIC > 2 μg/mL), Ciprofloxacin (MIC > 2 μg/mL), and Tetracycline (MIC > 4 μg/mL). Additionally, against Gram-negative bacteria *Pseudomonas aeruginosa*, the antibiotics Gentamycin (MIC ≥ 16 μg/mL), Tetracycline (MIC ≥ 16 μg/mL), and Ciprofloxacin (MIC ≥ 4 μg/mL) exhibited inhibitory activity. Therefore, Ampicillin was ineffective against *Pseudomonas aeruginosa*. Despite some antibiotics requiring higher inhibitory concentrations, this study highlights the significant potential of bull thistle ethanol and dry extracts. The findings suggest that bull thistle extracts, whether ethanolic or dry, should be used in concentrated formulations to maximize their effectiveness.

## 4. Discussion

Medicinal plants are vital to primary healthcare because they can be used to treat a variety of illnesses. Polyphenolic compounds are commonly present in various plants and have several biological functions, including antioxidant, antibacterial, and others [20]. Secondary metabolite biosynthesis, including phenolic compounds, is dynamic and influenced by various plant and environmental variables. The plant type and ontogenetic development phase are the most critical first-group characteristics. Polyphenolic compound concentration and accumulation vary among plant sections and organs, reflecting their function in the lifecycle and growth phases [21]. This study revealed the phytochemical composition of *C. vulgare* root, leaf, and inflorescence samples in different phenological stages, the plant parts of the collected raw materials, and antioxidant and antimicrobial effects. The accumulation of bioactive compounds in *C. vulgare* is influenced by various factors, including the growth years and specific plant parts.

During this research, it was determined that chlorogenic acid and apigenin-7-*O*-glucoside were the dominant phenolic compounds in all extracts of *C. vulgare*. Mainly, chlorogenic acid was detected in extracts from *C. vulgare* leaves, made from raw materials collected from the end of the phenological dormancy period on 23 May (yields 146.28 mg/g) and during the mass regrowth period on 6 June (yields 140.15 mg/g). Also, 64.083 mg/g chlorogenic acid was detected in inflorescence extracts made from raw materials collected the second growing year at the beginning of the flowering stage on 18 July. The least amount of chlorogenic acid was detected in the extract from *C. vulgare* roots, which was made from raw materials collected at the end of the phenological dormancy period stage on 9 May (yields 31.71 mg/g) during the second growing year. Apigenin-7-*O*-glucoside was detected in the extracts from *C. vulgare* leaves, which were made from raw materials collected at the end of the phenological dormancy period in the first growing year on 23 May; moreover, a large amount of apigenin-7-*O*-glucoside was detected in the extracts, which were made from leaves collected at the buttoning at both growing years on 4 July. The most significant amount of apigenin-7-*O*-glucoside was detected in extracts from *C. vulgare* leaves collected at the mass flowering phenological stage in the second growing year on 1 August (yields 147.96 mg/g). The lowest amount of apigenin-7-*O*-glucoside was detected in all extracts from *C. vulgare* roots from raw materials collected at different phenological states. Also not detected or detected in minimal quantities were apigenin-7-*O*-glucoside in the extracts from *C. vulgare* inflorescences, which were made from raw materials collected at the seed maturity and mass flowering phenological stages, but the maximal amount of apigenin-7-*O*-glucoside was detected in inflorescence samples collected at the buttoning stage of the first growing year on 4 July (yields 76.30 mg/g). Baldermann et al. researched sweet cherry buds (*Prunus avium* L.) and showed that the content of phenolic compounds decreases until the end of endodormancy [22]. However, Mendez-Lopez et al. indicated that polyphenols and flavonoids did not vary significantly in phenological stages (seed germination, development of trifoliolate leaves, formation of secondary shoots, elongation of the main stem, development of harvestable vegetative parts before the emergence of inflorescences, emergence of inflorescences, flowering, pod development, and maturation of pods) of chipilín (*Crotalaria longirostrata* Hook. & Arn.) extract [23]. Kozyra et al. detected chlorogenic acid in 50% methanolic, 80% methanolic, 100% methanolic, dichloromethane, acetone, and ethyl acetate inflorescence extracts obtained from *C. canum*. Also, they detected apigenin in the same extracts of *C. canum*, except dichloromethane extract [24]. Nazaruk et al. detected total phenolic content and antioxidant activity in MeOH extracts, CHCl_3_, Et_2_O, EtOAc, and BuOH fractions from *C. vulgare* inflorescences and leaves. Still, they only collected samples during full flowering from June to August [8]. Later, Nazaruk et al., for the first time, isolated apigenin-7-*O*-glucoside and other flavonoid compounds from methanolic extracts, which were fractionated Et_2_O, EtOAc, and n-BuOH of flowers of *C. rivulare*, but only collected flowers in June, which means during the mass regrowth phenological stage [25]. Hossain et al. also detected phenolic compounds in the ethanol extract of *C. arvense* aerial parts collected in February. They showed that extracts contained a moderate concentration of rutin hydrate and quercetin [26].

Significant amounts of phenolic compounds were discovered during the HPLC-PDA analysis. As a result, it was decided to examine the antioxidant properties of the extracts using the CUPRAC method. It was found that the active constituents derived from various growth years and organs of *C. vulgare* demonstrated diverse levels of antioxidant properties. The highest antioxidant activity was present in the extracts from the *C. vulgare* leaves collected during the first growing year at the end of the phenological dormancy period on 9 May (12,938 TE/g DW). However, the antioxidant activity decreased in the second growing year, totaling 5113 TE/g DW. Slightly lower antioxidant activity was determined in extracts from *C. vulgare* roots made from the raw materials of the first growing year collected at the end of the phenological dormancy period on 23 May (9213 TE/g DW). The antioxidant activity in extracts obtained from *C. vulgare* inflorescences collected at the seed maturity stage on 12 September was 8331 TE/g DW. Additionally, the antioxidant activity decreased in the second growing year, resulting in 2413 TE/g DW. Overall, the lowest antioxidant activity was detected in extracts from *C. vulgare* roots collected at the seed maturity stage in the second-growing year (238 TE/g DW). It is important to note that the relationship between antioxidant activity and total chlorogenic acid content for ethanolic extracts from *C. vulgare* was positive and highly significant. Correlation coefficients, R^2^, amounted to 0.62 and 0.75 in extracts made from leaves from the first and second growing years, respectively, 0.39 and 0.97 in extracts made from inflorescences, and 0.48 and 0.93 in extracts made from roots also from the first and second growing years, respectively. The relationship between antioxidant activity and total apigenin-7-*O*-glucoside content for ethanolic extracts from *C. vulgare* was positive only in leaf extracts (correlation coefficients R^2^ amounted to 0.20 and 0.65 at the first and second growing years, respectively). Therefore, our results show that the antioxidant activity is more dependent on the amount of chlorogenic acid. A series of investigations examining the antioxidant properties of inflorescences and foliage of *Cirsium* species cultivated in the north-eastern part of Poland revealed a positive correlation between the two and the overall phenolic content of the plants [8,27]. An unequivocal association has been documented between the overall polyphenol concentration and the ability to scavenge DPPH free radicals [28]. Kurç et al. found that the methanol extract of whole plant parts of *C. vulgare* (roots, stems, leaves, and inflorescences) collected in June had the maximum antioxidant activity by CUPRAC assay, with a Trolox equivalent antioxidant capacity of 2.14 mmol/g and an EC_50_ value of 18.5 μg/mL. Also, they detected that the same extract possesses the highest antioxidant activity by FRAP (1436.6 µmol Fe“2+”/g extract), ABTS (1436.6 µmol Fe“2+”/g extract), and superoxide anion radical scavenging activity assays. Moreover, they used the β-carotene-linoleic acid emulsion model. They detected that the compounds extracted by methanol appeared to have the most efficient antioxidant activity in the lipid system, similarly to the ABTS and FRAP activity results [11]. Demirtas et al. indicated that crude extracts from *Cirsium arvense* leaves collected in June possess the highest antioxidant activity by DPPH and FRAP assays [29]. Our results also showed that the highest antioxidant activity was possessed by extracts from *C. vulgare* leaves; therefore, it showed that maximum antioxidant activity was detected at the end of the phenological dormancy period (12,938 TE/g DW) and decreased at the beginning of mass flowering (8438 TE/g DW) and during the mass flowering (4737 TE/g DW) phenological stages during the first growing years. Naghiloo et al. also determined that the leaf methanolic extracts from *Astragalus compactus* Lam. possess the highest antioxidant activity compared with root and flower extracts. The maximal activity was determined in the fructification phenological stage [30]. Zhao et al. investigated methanol extracts from seven *Cirsium* species (*C. arisanense* Kitam., *C. brevicaule* A. Gray, *C. ferum* Kitam., *C. hosokawae* Kitam., *C. japonicum* DC. var. australe Kitam., *C. kawakamii* Hayata, and *C. lineare* (Thunb.) Sch. Bip.) materials, including the aerials, flowers, and radix parts collected from August to September [31]. They showed that methanol extract from *Cirsium japonicum* var. australe flower parts possessed the best antioxidant activity by DPPH, ABTS, and hypochlorite-ion-scavenging assays [31], while it showed that ethanol extract from *C. vulgare* leaves possessed the best antioxidant activity (12,938 TE/g DW), especially from raw materials collected at the end of the phenological dormancy period at the first growing year. However, ethanolic extracts from inflorescences also had high antioxidant activity; the highest was detected at the seed maturity phenological stage in the first growing year (8331 TE/g DW). Moghaddam et al. showed that ethanolic extracts from *Famaria vaillantii* aerial parts collected during the vegetative growth stage possess the highest antioxidant activity by DPPH and FRAP assays [32]. The vegetative stage means two main sequential periods: the dormancy period and the growing season [33], which partly corresponds to the end of the phenological dormancy period in this research. Different antioxidant activity assays revealed that the ethanol extract of *Cirsium arvense* exhibited dose-dependent antioxidant activity compared to the standard [26]. Yin et al. tested the antioxidant and antidiabetic activities of extracts from *Cirsium japonicum* roots. They showed that the MeOH extracts exhibited more potent free radical scavenging activity than the water extracts. Both extracts exhibited concentration-dependent hydroxyl radical scavenging activity and a reduced power and metal chelating capacity [34]. Kim et al. identified six polyphenolic compounds (syringin, chlorogenic acid, 3,5-di-caffeoylquinic acid, 3′-hydroxycinnamic acid, 3,4-dicaffeoylquinic acid, and oenin) in *Cirsium japonicum* extracts and confirmed their potential antioxidant effect [35]. It was found that *C. vulgare* extracts exhibited antioxidant properties, most likely due to chlorogenic acid.

Multidrug-resistant (MDR) microorganisms are a major danger to international health. Due to the rise of bacteria resistant to antibiotics, many of the illnesses to which we are exposed today are difficult to treat with current medications, which are rapidly losing their effectiveness. The identification of novel antibacterial drugs capable of combating antibiotic-resistant bacteria is a top priority in scientific research [36]. Plant secondary metabolites are appealing natural reservoirs for antibacterial compound research due to their chemical variety and extensive history of use [37]. Using medicinal plants to develop novel antibacterial medications has many advantages over synthetic or semisynthetic antibiotics: availability and cost-effectiveness, herbal medicines have few negative effects and interact safely with critical systems, the repertory of bioactive compounds with antibacterial activities produced by medicinal plants is broad and infinite, eco-friendly approaches can extract medicinal plant compounds with low pollution, and medicinal herbs have various complementary and synergistic effects, making them attractive antibiotic-resistant bacterium treatments [38]. There is data in the literature that chlorogenic acid, the highest amount of which was determined in *C. vulgare* leaves collected at the end of the phenological dormancy period and the mass regrowth ethanolic extracts, possesses antimicrobial effects, especially against *Escherichia coli* [39]. Moreover, the flower head and leaf extracts from *C. palustre*, *C. arvense, C. oleraceum*, *C. rivulare,* and *C. vulgare* possess antimicrobial activity with a 10 mg/mL MIC value, and this effect is due to chlorogenic acid [27,40,41]. The antibacterial activity of the *Cirsium* plant is attributed to its phenolic acids. The phenolic hydroxyl group can cause metal complex reactions in bacteria, destroying their nutritional substrate and resulting in antibiotic activity. Moreover, by inhibiting ergosterol production, respiration, succinate dehydrogenase (SDH), and NADH oxidase, phenolic acids have antifungal properties [42]. The extracts with the highest phenolic compounds and antioxidant activity were selected for further studies. This research showed that ethanolic extracts from *C. vulgare* had no antimicrobial effect against *Bacillus cereus* and *Escherichia coli*. Kenny et al. demonstrated that neither water nor ethanolic extracts from *C. vulgare* had any antimicrobial effect against *Staphylococcus aureus*, MRSA, *Bacillus cereus*, *Escherichia coli*, and *Salmonella typhi* [43]. However, it was demonstrated that ethanolic extracts, which were made from leaves collected at the end of the phenological dormancy period on 23 May (U13), had an antimicrobial effect against *Staphylococcus aureus*. Additionally, it was shown that the ethanolic extracts from *C. vulgare* leaves collected at the end of the phenological dormancy period on 25 April and 23 May (U10 and U13) and the extract from leaves collected during the mass regrowth period on 6 June (U12) had an antimicrobial effect against *Staphylococcus epidermidis*. The ethanolic extract from *C. vulgare* leaves collected at the end of the phenological dormancy period on 25 April (U10) had antimicrobial activity against *Pseudomonas aeruginosa*. Moreover, the ethanolic extracts from leaves collected at the end of the phenological dormancy period on 25 April (U4, U10) and on 23 May (U11) and mass regrowth period on 6 June (U12), and extracts from roots collected at the end of the phenological dormancy period on 11 April (U1) had antimicrobial activity against *Proteus vulgaris*. Also, investigation showed that ethanolic extracts from *C. vulgare* leaves collected at the end of the phenological dormancy period on 25 April (U4, U10) and on 23 May (U5, U11) and during the mass regrowth period on 6 June (U6, U12) and seed maturity period on 12 September possessed an antifungal effect against *Candida albicans*. In addition, extracts from *C. vulgare* inflorescences collected during the mass flowering period on 30 July (U7) and seed maturity period on 12 September (U9) also showed antifungal activity against *Candida albicans.* The widest antimicrobial activity (against *Staphylococcus epidermidis*, *Pseudomonas aeruginosa*, *Proteus vulgaris,* and *Candida albicans*) was shown by the extract made from *C. vulgare* leaves collected at the end of the phenological dormancy period on 25 April (U10). It is crucial to note that dry extracts from *C. vulgare* inflorescences collected during the seed maturity period of the first growing year on 12 September and from roots collected at the end of the phenological dormancy period during the second growing year on 11 April (UF, UR), unlike ethanolic extracts, had antimicrobial concentration-dependent effects against *Bacillus cereus*. Also, these extracts had concentration-dependent antimicrobial effects against *Staphylococcus aureus*, *Staphylococcus epidermidis,* and *Proteus vulgaris,* as well as the ethanolic extracts as studied. It is important to note that drying of extracts increased antimicrobial potential in our opinion, due to the concentration of biologically active compounds. Ethanolic extracts from *C. vulgare* roots collected at the end of the phenological dormancy period possessed antimicrobial activity against *Proteus vulgaris* and *Candida albicans*. Meanwhile, dry extracts from the same aerial parts possessed antimicrobial activity against *Staphylococcus aureus*, *Staphylococcus epidermidis*, *Bacillus cereus*, and *Proteus vulgaris*. Moreover, ethanolic extracts from *C. vulgare* inflorescences, collected at the seed maturity period, possessed antimicrobial activity only against *Candida albicans*. Meanwhile, dry extracts possessed antimicrobial activity against *Staphylococcus aureus*, *Staphylococcus epidermidis*, *Bacillus cereus* and *Proteus vulgaris*. The widest antimicrobial activity was possessed by dry extracts from *C. vulgare* inflorescences collected during the seed maturity phenological stage and from roots collected at the end of the phenological dormancy period, especially in 1 mL and 0.5 mL amounts. Our findings agree with the study of Borawska et al., who examined *C. vulgare* and other *Cirsium* species extracts [44]. Evidence has demonstrated that inflorescences and leaf dry extracts derived from *C. vulgare* and other *Cirsium* species have notable antibacterial activity against *Pseudomonas aeruginosa* and *Staphylococcus aureus*. Additionally, they showed that *Cirsium* species extracts have antimicrobial effects against *Bacillus subtilis*. The antibacterial effect of the flower extract from *Cirsium* species was stronger than that of the leaf extract [44]. Kozyra et al. showed that *C. canum* (L.) All. inflorescence extracts possess antimicrobial activity against *Staphylococcus aureus*, *S. pneumoniae*, *Streptococcus pyogenes*, *S. epidermidis*, *Streptococcus mutans*, *M. luteus*, *Bacillus cereus,* and *B. subtilis*. The maximal antimicrobial activity showed extracts richer in phenolic compounds [24]. Kurç et al. showed that methanol, ethyl acetate, diethyl ether, and hexane extracts of whole plant parts of *C. vulgare* (root, stem, leaf, and inflorescence) collected in June had antibacterial activity against *S. aureus*, *B. subtilis*, *E. coli*, *P. aeruginosa*, *P. mirabilis*, *S. typhimurium,* and antifungal activity against *C. albicans*, *C. glabrata*, *C. parapsilosis*, *C. krusei*, *P. chrysogenum*, and *A. fumigatus*. The highest antibacterial activity was detected in ethyl acetate and diethyl ether extracts. Meanwhile, the highest antifungal activity was detected in hexane extracts. Also, they demonstrated that the methanol extract of *C. vulgare* possesses antimicrobial activity against *S. aureus*, *P. aeruginosa,* and *C. albicans* [11]. Also, there are data about the flavonoid apigenin-7-*O*-glucoside extracted from *C. arvense* whole grass, which provides antimicrobial activity together with other flavonoids [45]. The antibacterial action of flavonoids may be linked to their polarity and lipid–water distribution coefficient. Lipophilic flavonoids increase activity, reduce cell fluidity, and inhibit Gram-positive bacteria by long acyl membrane binding [42]. Although ethanolic extracts from *C. vulgare* leaves and dry extracts from *C. vulgare* inflorescences and roots had weaker effects against *Staphylococcus aureus*, *Staphylococus epidermidis,* and *Pseudomonas aeruginosa* compared to known antibiotics Ampicillin, Gentamicin, Tetracycline, and Ciprofloxacin, the findings from these experiments indicate that medicinal plants, such as *C. vulgare*, can potentially serve as viable substitutes for synthetic formulations. Consequently, a comprehensive evaluation of their antimicrobial properties was warranted.

## 5. Conclusions

This study is unique in being the first to examine the antioxidant and antimicrobial effects of extracts from leaves, inflorescences, and roots of *C. vulgare*, collected at various phenological stages over two growing years. Based on the initial chemical investigation, it is most likely that chlorogenic acid and apigenin-7-*O*-glucoside are the main components of the substance. The extracts from *C. vulgare* leaves, collected at the end of the phenological dormancy period and the first growing year, exhibited the most significant antioxidant and antimicrobial activity. These extracts contained the highest concentration of chlorogenic acid and a significant amount of apigenin-7-*O*-glucoside. The lowest antioxidant activity was determined in *C. vulgare* ethanolic extracts from roots collected at the seed maturity stage. Furthermore, chlorogenic acid and apigenin-7-*O*-glucoside were detected in small quantities in root extracts. Drying the extracts from *C. vulgare* inflorescences collected during the seed maturity period and roots collected during the phenological dormancy period concentrated the active compounds and caused antimicrobial activity. This finding has important implications for the food and pharmacy industries in terms of selecting phenological stages and raw material parts that have high antioxidant potential and a high concentration of phenolic compounds, all of which can be used to produce specialized antioxidants that promote health.

## Figures and Tables

**Table 1:** Antimicrobial effect of *C. vulgare* ethanolic extract in Muller–Hinton agar (5 mL) on the growth of reference microorganism cultures. Extract number meaning: U1: roots from the end of the phenological dormancy period (11 April 2022), U2: roots from the end of the phenological dormancy period (25 April 2022), U3: roots from the end of the phenological dormancy period (9 May 2022), U4: leaves from the end of the phenological dormancy period (25 April 2022), U5: leaves from the end of the phenological dormancy period (23 May 2022), U6: leaves from the mass regrowth period (6 June 2022), U7: inflorescences from the mass flowering period (30 July 2021), U8: leaves from the seed maturity period (12 September 2021), U9: inflorescences from the seed maturity period (12 September 2021), (0.3 g raw materials + 30 mL ethanol); U10: leaves from the end of the phenological dormancy period (25 April 2022), U11: leaves from the end of the phenological dormancy period (23 May 2022), U12: leaves from the mass regrowth period (6 June 2022) (1 g raw materials + 20 mL ethanol); U13: leaves from the end of the phenological dormancy period (23 May 2022) (2 g raw materials + 10 mL ethanol); U13*: leaves from the end of the phenological dormancy period (23 May 2022) (2 g raw materials + 10 mL ethanol).

Ethanol Extracts of *C. vulgare* (mL)															
Reference Cultures of Microorganisms	U1 1.0	U2 1.0	U3 1.0	U4 1.0	U5 1.0	U6 1.0	U7 1.0	U8 1.0	U9 1.0	U10 1.0	U11 1.0	U12 1.0	U13 1.0	U13* 0.5	Ethanol 1.0
*Staphylococcus aureus* ATCC 25923	NA	NA	NA	NA	NA	NA	NA	NA	NA	NA	NA	NA	8.35	NA	NA
*Staphylococcus epidermidis* ATCC 12228	NA	NA	NA	NA	NA	NA	NA	NA	NA	16.7	NA	8.35	8.35	NA	NA
*Enterococcus faecalis* ATCC 29212	NA	NA	NA	NA	NA	NA	NA	NA	NA	NA	NA	NA	NA	NA	NA
*Escherichia coli* ATCC 25922	NA	NA	NA	NA	NA	NA	NA	NA	NA	NA	NA	NA	NA	NA	NA
*Klebsiella pneumoniae* ATCC 13883	NA	NA	NA	NA	NA	NA	NA	NA	NA	NA	NA	NA	NA	NA	NA
*Pseudomonas aeruginosa* ATCC 27853	NA	NA	NA	NA	NA	NA	NA	NA	NA	16.7	NA	NA	NA	NA	NA
*Bacillus cereus* ATCC 11778	NA	NA	NA	NA	NA	NA	NA	NA	NA	NA	NA	NA	NA	NA	NA
*Proteus vulgaris* ATCC 8427	16.7	NA	NA	16.7	NA	NA	NA	NA	NA	16.7	8.35	8.35	NA	NA	NA
*Candida albicans* ATCC 10231	16.7	NA	16.7	16.7	16.7	16.7	16.7	16.7	16.7	16.7	8.35	8.35	NA	NA	NA

NA—does not inhibit the growth of the reference culture of microorganisms (no antimicrobial effect); MIC values are presented mg/mL.

**Table 2:** Antimicrobial effect of *C. vulgare* dry extract in Muller–Hinton agar (5 mL) on the growth of reference microorganism cultures. Extract letter meanings: UF-*C. vulgare* inflorescences from the seed maturity period (first year 12 September), UR-*C. vulgare* roots from the end of the phenological dormancy period (second year 11 April).

Amount of *C. vulgare* Extract (mL)												
Reference Cultures of Microorganisms	UF 1.0	UF 0.5	UF 0.4	UF 0.3	UF 0.2	UF 0.1	UR 1.0	UR 0.5	UR 0.4	UR 0.3	UR 0.2	UR 0.1
*Staphylococcus aureus* ATCC 25923	5.57	3	NA	NA	NA	NA	5.57	3	NA	NA	NA	NA
*Staphylococcus epidermidis* ATCC 12228	5.57	3	NA	NA	NA	NA	5.57	3	2.47	NA	NA	NA
*Enterococcus faecalis* ATCC 29212	NA	NA	NA	NA	NA	NA	NA	NA	NA	NA	NA	NA
*Escherichia coli* ATCC 25922	NA	NA	NA	NA	NA	NA	NA	NA	NA	NA	NA	NA
*Klebsiella pneumoniae* ATCC 13883	NA	NA	NA	NA	NA	NA	NA	NA	NA	NA	NA	NA
*Pseudomonas aeruginosa* ATCC 27853	NA	NA	NA	NA	NA	NA	NA	NA	NA	NA	NA	NA
*Bacillus cereus* ATCC 11778	5.57	3	NA	NA	NA	NA	5.57	NA	NA	NA	NA	NA
*Proteus vulgaris* ATCC 8427	5.57	NA	NA	NA	NA	NA	5.57	3	NA	NA	NA	NA
*Candida albicans* ATCC 10231	NA	NA	NA	NA	NA	NA	NA	NA	NA	NA	NA	NA

NA—does not inhibit the growth of the reference culture of microorganisms (no antimicrobial effect); All MIC values are presented mg/mL.

## Data Availability

All data generated during this study are included in this article.

## Appendix A

**Table A1:** Extracts numeration, parts of the plant, phenological stages, collection date, and type of extract.

Extract Number	Plant Part	Phenological Stage	Collection Date	Extract Type
U1	Roots	end of the phenological dormancy	11 April 2022	Ethanolic 50% (*v*/*v*)
U2	Roots	end of the phenological dormancy	25 April 2022	Ethanolic 50% (*v*/*v*)
U3	Roots	end of the phenological dormancy	9 May 2022	Ethanolic 50% (*v*/*v*)
U4	Leaves	end of the phenological dormancy	25 April 2022	Ethanolic 50% (*v*/*v*)
U5	Leaves	end of the phenological dormancy	23 May 2022	Ethanolic 50% (*v*/*v*)
U6	Leaves	mass regrowth period	6 June 2022	Ethanolic 50% (*v*/*v*)
U7	Inflorescences	mass flowering	30 July 2021	Ethanolic 50% (*v*/*v*)
U8	Leaves	seeds maturity	12 September 2021	Ethanolic 50% (*v*/*v*)
U9	Inflorescences	seeds maturity	12 September 2021	Ethanolic 50% (*v*/*v*)
U10	Leaves	end of the phenological dormancy	25 April 2022	Ethanolic 50% (*v*/*v*)
U11	Leaves	end of the phenological dormancy	23 May 2022	Ethanolic 50% (*v*/*v*)
U12	Leaves	mass regrowth period	6 June 2022	Ethanolic 50% (*v*/*v*)
U13	Leaves	end of the phenological dormancy	25 April 2022	Ethanolic 50% (*v*/*v*)
U13*	Leaves	end of the phenological dormancy	25 April 2022	Ethanolic 50% (*v*/*v*)
UF	Inflorescences	seeds maturity	12 September 2021	Dry
UR	Roots	end of the phenological dormancy	11 April 2022	Dry

**Table A2:** Validation data of HPLC-PDA analysis.

Active Compounds	Limit of Detection	Limit of Quantification	Range	R^2^	Repeatability	Accuracy	Formula
Chlorogenic acid	0.03 μg/mL	0.11 μg/mL	14.5–0.113 μg/mL	0.9999	0.21%	0.32%	3.14e + 004X + 5.89e + 001
Apigenin-7-*O*-glucoside	0.320 μg/mL	0.078 μg/mL	20.0–0.625 μg/mL	0.9999	0.41%	0.63%	3.53e + 004X − 1.41e + 002
Apigenin	0.063 μg/mL	0.200 μg/mL	16.10–0.250 μg/mL	0.9999	0.12%	0.20%	3.42e + 004X − 1.59e + 003
Luteolin	0.053 μg/mL	0.195 μg/mL	110–0.214 μg/mL	0.9999	0.1%	0.15%	4.23e + 004X + 3.89e + 002
Hyperoside	0.132 μg/mL	0.264 μg/mL	16.9–0.528 μg/mL	0.9997	0.47%	1.02%	2.13e + 004X + 5.81e + 002
Neochlorogen	0.035 μg/mL	0.105 μg/mL	36–0.141 μg/mL	0.9999	0.15%	0.42%	1.33e + 004X − 4.08e + 002
Isoquercitrin	0.109 μg/mL	0.217 μg/mL	27.85–0.870 μg/mL	0.9999	0.45%	0.86%	2.14e + 004X + 2.09e + 003
p-coumaric acid	0.123 μg/mL	0.404 μg/mL	103.50–0.81 μg/mL	0.9999	0.32%	0.63%	7.59e + 004X + 9.76e + 003
Caffeic acid	0.318 μg/mL	1.020 μg/mL	2.554–81.750 μg/mL	0.99993	0.3%	0.4%	5.83 + 004X + 3.36e + 004
trans-ferulic acid	0.345 μg/mL	1.104 μg/mL	5.521–176.700 μg/mL	0.99980	0.2%	0.3%	5.18e + 004X − 2.08e + 004
4-*O*-Caffeoilquinic acid	0.135 μg/mL	0.779 μg/mL	0.859–25.500 μg/mL	0.999	0.1%	0.3%	5.88e + 004X + 9.89e + 003

**Table A3:** The antimicrobial activity (MIC) of C. vulgare ethanol and dry extracts and antibiotics n = 3.

Compound Name	Reference Cultures of Microorganisms		
	*Staphylococcus aureus* ATCC 25923	*Staphylococcus epidermidis* ATCC 12228	*Pseudomonas aeruginosa* ATCC 27853
U10*	NA	16.7 mg/mL	16.7 mg/mL
U12*	NA	8.35 mg/mL	NA
U13*	8.35 mg/mL	8.35 mg/mL	NA
UF1.0	5.57 mg/mL	5.57 mg/mL	NA
UF0.5	3 mg/mL	5.57 mg/mL	NA
UR1.0	5.57 mg/mL	5.57 mg/mL	NA
UR0.5	3 mg/mL	3 mg/mL	NA
UR0.4	NA	2.47 mg/mL	NA
Ampicillin	≥0.5 μg/mL	≥0.5 μg/mL	NA
Gentamycin	≥2 μg/mL	≥2 μg/mL	≥16 μg/mL
Tetracycline	≥4 μg/mL	≥4 μg/mL	≥16 μg/mL
Ciprofloxacin	≥2 μg/mL	≥2 μg/mL	≥4 μg/mL

NA—no activity; extracts did not inhibit the reference microbial strains in their ≤16.7 mg/mL concentration; Compound name meaning: U10*–U12* ethanolic extracts from *C. vulgare* leaves, UF-UR dry extracts from *C. vulgare* inflorescences and roots; (*) The antimicrobial activity was evaluated against solvent. The solvent (50% (*v*/*v*) ethanol) did not inhibit the reference microbial strains.
